# Dietary Fibre Supplementation Improves Semen Production by Increasing Leydig Cells and Testosterone Synthesis in a Growing Boar Model

**DOI:** 10.3389/fvets.2022.850685

**Published:** 2022-03-11

**Authors:** Yan Lin, De Wu, Lianqiang Che, Zhengfeng Fang, Shengyu Xu, Bin Feng, Yong Zhuo, Jian Li, Caimei Wu, Junjie Zhang, Lujie Li

**Affiliations:** ^1^Key Laboratory of Animal Disease-Resistance Nutrition and Feed Science, Ministry of Agriculture, Sichuan Agricultural University, Chengdu, China; ^2^Key Laboratory of Animal Disease-Resistance Nutrition, Ministry of Education, Chengdu, China; ^3^College of Life Science, Sichuan Agricultural University, Ya'an, China

**Keywords:** fibre, spermatogenesis, Leydig cells, testosterone, boar

## Abstract

Testicular development is imperative to spermatogenesis, and pre-puberty is the key period for testis development. This study, therefore, investigated the effects of fibre supplementation on testis development and its possible mechanism in a growing boar model. Thirty Yorkshire boars were randomly divided into a control group (Control) and a fibre group (Fibre) from day 0 to 90 after weaning, with three pigs per pen and five pens per treatment. Blood and testes were collected for analysis. Dietary fibre supplementation had no significant effect on growth performance, testicular volume, or libido but increased the semen production of boars. Boars fed with fibre had lower serum cholesterol (CHO) and low-density lipoprotein (LDL) levels compared to those on the Control diet; however, testicular CHO, triglyceride (TG), and LDL concentration in the Fibre group were significantly higher than the Control group (*P* < 0.01). Testicular histological analysis showed that seminiferous tubules and testicular germ cells of 120-day-old boars were densely arranged in the Fibre group, and the number of Leydig cells was significantly higher than that of the Control group (*P* < 0.001). Furthermore, the diet supplemented with fibre significantly decreased leptin, leptin receptor (Leptor), and luteinising hormone (LH) concentrations in boar serum (*P* < 0.05), whereas follicle-stimulating hormone (FSH) and testosterone concentrations were significantly increased (*P* < 0.05). Meanwhile, the expression of *AMH, AMHR2*, and *SYCP3* genes related to proliferation and differentiation, and hormone-related genes *STAR* and *SOCS3*, were significantly up-regulated (*P* < 0.05). *OCCLUDIN* expression was up-regulated, whereas *CDH2* expression was down-regulated. In conclusion, increased fibre intake during the pre-puberty period in growing boar is crucial for Leydig cell proliferation, up-regulating the expression of genes related to hormone synthesis and thereby promoting the secretion of testosterone and semen production.

## Introduction

The testis is an important sexual organ in male animals. In humans and other male mammals, such as rats and boars, testicular development directly affects semen quality (1, 2), which is crucial for reproduction. After birth, testicular development mainly occurs in the seminiferous tubules, including the proliferation of Sertoli, germ, and Leydig cells. Human testes mainly develop during childhood (2–11 years) and adolescence (2–14 years) (3). From 2–8 years of age, the number of germ cells increases by 3 to 6 times, and testis volume, testis weight, Sertoli cells, and testosterone levels in the testes gradually increase (4); subsequently, the spermatogonial cells proliferate rapidly. Testicular development of boars is similar to that of humans and can be divided into two key stages. During the pre-puberty period, the number of germ cells increases, and boars nearly reach sexual maturity at approximately 120–150 days. Therefore, testicular development during the growing period is very important for later spermatogenesis.

As an important nutrient, it is believed that fibre intake may have beneficial effects on reproduction and health. However, there is still little direct evidence of its effect on testicular development and spermatogenesis. It has been reported that sperm concentration and motility in humans are positively influenced by cereal consumption, and vegetables and fruits also have a great effect on sperm motility. These substances contain a higher level of dietary fibre (5). Studies in rabbits have shown that the addition of dietary fibre (hemicellulose and soluble fibre) with moderate feed intake restriction decreased sperm abnormalities and increased the percentages of normal and motile spermatozoa and the insemination ratio (6). Increased fibre intake can improve sex hormone-binding globulin levels, thereby affecting the activity of related sex hormones involved in testicular development. The dietary pattern, which was high in vitamins, dietary fibre, and polyunsaturated fatty acids, was significantly associated with a lower risk of asthenozoospermia (7). Our previous study in mice and pigs demonstrated that fibre supplementation regulates animal reproduction by affecting hormone secretion and nutrient metabolism (8) and that dietary fibre supplementation during pregnancy improved testicular development in the offspring boars (9). The above results suggest that fibre plays a positive role in boar reproduction and may be a useful nutrient for improving male fecundity. As we know, testicular development of boars before sexual maturity is very important for spermatogenesis in adulthood. The decrease of germ cell numbers induced by Bisphenol A in mice or heat stress is accompanied by the decrease in spermatogenesis (10, 11). However, it is not clear whether supplementary fibre during pre-puberty benefits testicular development and semen production in boars. Therefore, growing boars were used as an experimental animal model in this study to investigate the effect of fibre supplementation on testicular development and possible mechanisms.

## Materials and Methods

### Experimental Animals and Design

Thirty healthy Yorkshire purebred boars with similar body weights (8 ± 0.46 kg) were selected and randomly divided into two groups: control (Control) and fibre (Fibre). There were three pigs per pen and five pens per treatment. Feed was prepared according to the principle of equal energy and nitrogen, with inulin and wheat bran as fibre sources. The diet formula was prepared according to the NRC 2012 (the nutritional levels of the diet are shown in Table 1).

**Table 1:** Nutritional levels of the experimental diet.

**Material and composition (%)**	**0–30 day**		**30–90 day**	
	**Control**	**Fibre**	**Control**	**Fibre**
**Nutrient level**
DE Mcal/kg	3.54	3.54	3.45	3.45
CP%	18.81	18.81	15.68	15.68
CF%	1.60	1.89	2.19	2.57
SF%	1.72	1.84	1.76	2.43
ISF%	8.25	9.63	10.92	12.75
TDF%	9.97	11.47	12.68	15.18
Ca%	0.81	0.81	0.68	0.68
AP%	0.48	0.48	0.34	0.34

*DE, digestible energy; AP, Available phosphorus; SF, Soluble fibre; ISF, Insoluble fibre; TDF, Total dietary fibre*.

Animal experiments were approved by the Sichuan Agricultural University under the Ethics Approval Code SCAUAC201906-03. Boars were raised in the teaching and scientific research farm and fed at 08:00, 14:00, and 20:00 each day, with free access to food and water. The initial and final body weights were recorded, and the feed intake was recorded daily. Other routine care, such as disinfection and immunity, was performed according to farm requirements.

After 120 days, the boars were fed the same diet and then trained at 6.5 months of age. Semen was collected once a week at 8 months for semen quality analysis.

### Sample Collection

At 120 days of age, six boars in each group were fasted for 12 h with unlimited access to drinking water, followed by blood collection through the anterior vena cava. The blood sample was placed in a 1.5 ml EP tube for 30 min and then centrifuged for 10 min at a speed of 3,000 rpm. The supernatant was collected and stored at −20°C. Five boars in each group were selected for testis sampling following anaesthetization. The testes were removed and weighed. Testicular tissues with a size of 1 × 1 × 1 cm were selected and stored in 4% paraformaldehyde for 24 h for histological analysis. Other testicular tissues were stored at −80°C.

### Growth Performance and Sperm Quality

Body weights and feed intake of the boars were recorded, and the average daily gain and average daily feed intake of each boar was calculated. Once a month, the testicular length, width, and thickness were measured with a Vernier calliper, and testicular volume was calculated using the ellipsoid formula.

Ejaculation response time and ejaculation duration were also measured (12). A computer-assisted sperm analysis (CASA) system was used to determine sperm motility and density. Total spermatozoa and effective spermatozoa per ejaculation were measured in reference to the calculation formula (13).

### Analysis of Serum and Testicular Hormone and Metabolite Contents

Approximately 200 mg of testicular tissue was homogenised in a 2 mL centrifugal tube with 200 μL phosphate buffer. The supernatant was collected after centrifugation at 10,000 rpm for 30 min. The CHO, TG, HDL, and LDL contents of the testis supernatant and serum were detected using an automatic biochemical analyser (Hitachi 3100, Japan). The leptin, leptin receptor (Leptor), testosterone (T), FSH, and LH levels were determined using an enzyme-linked immunosorbent assay (ELISA) kit (Jiancheng Bio, Nanjing, China). Hormone detection was performed using the ELISA double-antibody sandwich method. The substrate TMB was used for colour development. There was a positive correlation between the colour and hormone concentration in the sample. The absorbance (OD) was measured by the microplate reader at a 450 nm wavelength.

### Histological Examination of Testis Tissues

Testis fixed in 4% paraformaldehyde were embedded in paraffin, cut into 5-μm-thick slices, stained with haematoxylin and eosin after wax removal, and sealed with neutral gum. Five histological slides from each boar and 10 section per slide were counted morphometrically, and processed using an optical microscope with the software programme Image-Plus 6.0(Media Cybernetics Inc., USA). The number of Sertoli cell or Leydig cell was counted morphometrically and calculated as the number of per seminiferous tubule. Finally, a total of 500 cross-sections of seminiferous tubules per group were counted.

### Gene Expression

Gene expression in the testes was assayed by fluorescence-based quantitative PCR. The primer information is shown in Table 2. Total RNA was extracted from 50 mg of testicular tissue using a total RNA extraction kit (Tianmo Biotech Co., Ltd., China). Reverse transcription was performed using the PrimeScript™ RT Reagent Kit with gDNA Eraser (TaKaRa, Japan). Fluorescence-based quantitative PCR (qRT-PCR) assays were performed using an ABI7900HT fast, real-time PCR system (Applied Biosystems, USA) with a 20 μL reaction volume of SYBR Green PCR Master Mix (TaKaRa). qPCR conditions were as follows: 94°C for 30 s, followed by 40 cycles of 94°C for 5 s, and 60°C for 30 s. Each sample was analysed in triplicate. The mRNA expression levels were normalised to β-actin and calculated using the 2–^ΔΔCt^ method (14).

**Table 2:** Details of the primers.

**Gene name**	**(5^′^ → 3^′^) Sequence**	**Accession no**	**Product length (bp)**
*LePtin-F*	CGCGTCTATAAGAGGGGAGG	XM_021078503	98
*LePtin-R*	CCTTTCCGGGATGTGCTTCT		
*LePR-F*	GTGCTCGTGAATGTTGTGAATGCC	XM_021093493.1	127
*LePR-R*	GGCTGAACTGACATGAGAGGTGAG		
*LHR-F*	TGAAACACTTTCAAAATCCAGAGC	XM_021085887	103
*LHR-R*	GGTGCCATGCAGGTGAAATC		
*FSHR-F*	CTCACAGAATGATGTCTTAGAAGTG	XM_021085881	89
*FSHR-R*	GGTTGTTGGCCTTTTCAATTATTTC		
*KISS1-F*	TTCTTTGGGCCACTTCCTTCAAGG	XM_005656644	140
*KISS1-R*	GGGCTTTCTCTCCGCACACC		
*PDE3B-F*	ATGGCTCTATACGTGGCAGC	XM_021083299	85
*PDE3B-R*	GAGGGGCATTTGTAGCCACT		
*JAK2-F*	CTGGGTAGCCAATCCCCCTC	XM_021082799	193
*JAK2-R*	AGCCCTATTTACGTGCGACTC		
*STAT3-F*	GTGGAGAAGGACATCAGCGGTAAG	NM_001044580	149
*STAT3-R*	AGGTAGACCAGCGGAGACACAAG		
*SOCS3-F*	CCAGTACGATGCCCCACTTT	XM_021066253	95
*SOCS3-R*	TCTTGTGCTTGTGCCATGTG		
*STAR-F*	CTCAGCATTGACCTCAAGGGATGG	NM_213755	90
*STAR-R*	TCGCAGGTGATTGGCAAAGTCC		
*CYP11A1-F*	TTCGCTTCGCCTTTGAGTCCATC	XM_021098320	85
*CYP11A1-R*	TGAGCCTCAGGGTCCACTATTTCC		
*GDNF-F*	CACTGACTTGGGTTTGGGCTACG	XM_021076772	89
*GDNF-R*	CATACATGGTCTCGGCGGCATC		
*WT1-F*	ACATTCACTGCTCACCTGGA	XM_013994456	93
*WT1-R*	AGATACGGTCCTCCCTCCAC		
*AMH-F*	ATGTTTAGGGCAGCAGGCAA	XM_021081532	114
*AMH-R*	CAACGCCAGGAAGCCAGAGTC		
*AMHR2-F*	ACCACATTGTCCGTTTCATCAC	XM_021090944	240
*AMHR2-R*	TCAGATCTCGGTGGGCAATAC		
*AR-F*	ATTGTGTAAGGCAGTGTCGGTGTC	XM_021079513	121
*AR-R*	AGATGGTGGACCTGTCAGGAGTG		
*HSD3B1-F*	CATCCACACCAGCAGCATAGAGG	XM_021088745	149
*HSD3B1-R*	TAGCCTCCAGCACAGCCTTCTC		
*THP1-F*	GGGCAGCCTGAAGAGTAGAAAACG	XM_003133647	82
*THP1-R*	GGAGAGCCGCTGGTGGAGTC		
*CDH1-F*	CTGCTGCTCCTGCTCCTTATTCG	NM_001163060	123
*CDH1-R*	CTGGTCCTCTTCTCCACCTCCTTC		
*SYCP3-F*	ACCGGGAAATCTGGAAAGCCA	XM_003126677	102
*SYCP3-R*	GACATCCTCCTCTGAACCACTCA		
*CTNNB1-F*	AGACCGTGGAGGTAATGTGC	XM_013981492	121
*CTNNB1-R*	CTGCCCCTATGCAAGTCGAG		
*ZO-1-F*	CCAGGGAGAGAAGTGCCAGTAGG	XM_021098856	92
*ZO-1-R*	TTTGGTGGGTTTGGTGGGTTGAC		
*CDH2-F*	GGAGGCGGAGACTTGTGAAA	XM_021096205	85
*CDH2-R*	ATGGCCCAGCATTTGGATCA		
*OCLN-F*	TGGCTGCCTTCTGCTTCATTGC	XM_005672522	131
*OCLN-R*	GAACACCATCACACCCAGGATAGC		
*β-actin-F*	TCTGGCACCACACCTTCT	XM_021086047.1	114
*β-actin-R*	TGATCTGGGTCATCTTCTCAC		

### Statistical Analysis

Data was expressed as mean ± standard error and analysed using the SAS 9.4 software (SAS Institute Inc., Cary, NC, USA). If the measurement data conformed to the normal distribution, the independent sample *t*-test was used for analysis between the Control group and the Fibre group; otherwise, the non-parametric test was used. The level of statistical significance was set at *P* < 0.05.

## Results

### Growth and Testicular Development of Growing Boars

There were no significant differences in the average daily gain, average daily feed intake, feed conversion ratio, and testicular volume at each age between the Control and Fibre groups (*P* > 0.05), as shown in Table 3.

**Table 3:** Effects of dietary fibre supplementation on growth and development of reserve boars.

**Items**	**Control**	**Fibre**	***P*-value**
Initial body weight (kg)	8.80 ± 0.29	8.90 ± 0.40	0.855
Final body weight (kg)	60.59 ± 0.90	61.86 ± 1.59	0.496
Average daily gain (g)	665.47 ± 36.16	682.47 ± 24.35	0.504
Average daily feed intake (kg)	1.37 ± 0.033	1.32 ± 0.07	0.552
Feed conversion ratio	1.89 ± 0.048	1.78 ± 0.11	0.404
**Testicular volume (cm** ^**3**^ **)**			
8 WK.	15.86 ± 0.82	16.23 ± 0.58	0.709
10 WK.	36.93 ± 2.19	36.97 ± 2.59	0.991
13 WK.	123.00 ± 11.25	137.90 ± 8.08	0.291

*Data were expressed as the mean ± SEM*.

### Sexual Desire and Spermatogenesis in Adult Boars

**Figure 1:**
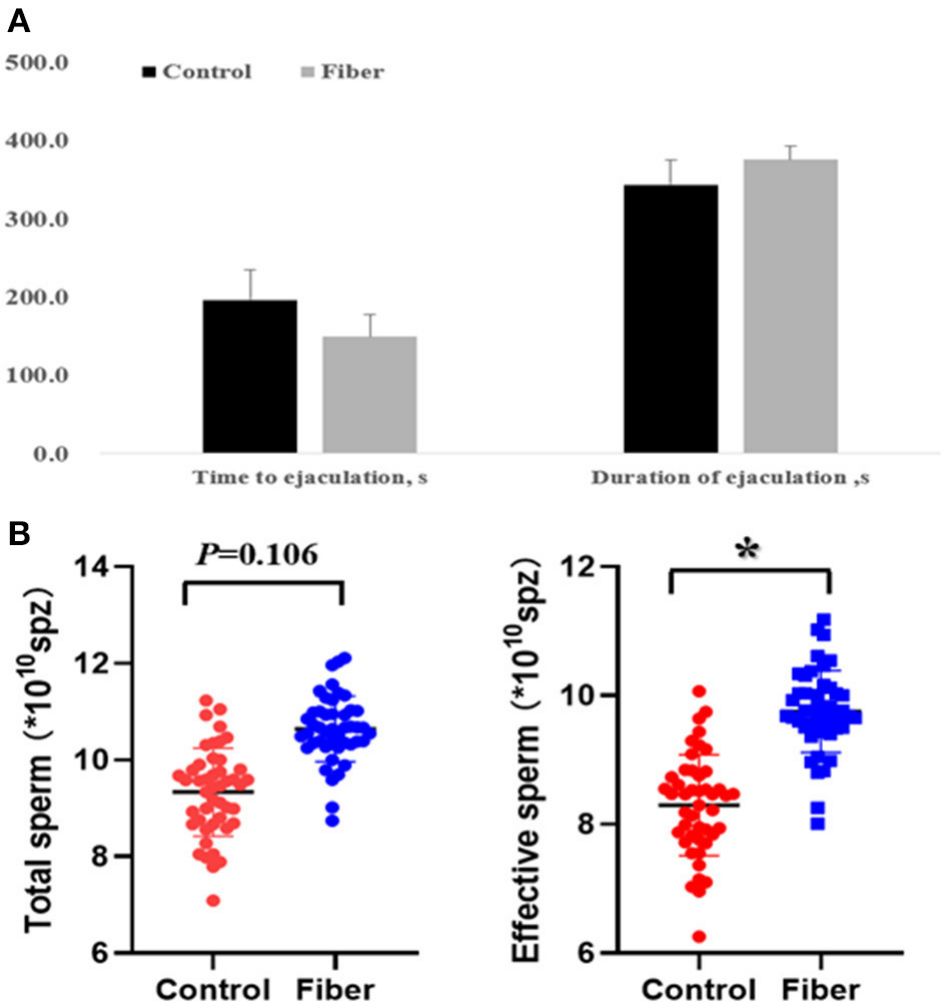
Effects of dietary fibre supplementation on libido and semen production of boars. **(A)** The boar libido performance, and data were expressed as the mean ± SEM. **(B)** The total sperm production and effective sperm production of boars. Data were expressed as the mean ± SEM. ^*^P < 0.05.

Semen was collected eight times to track the effect of fibre supply before sexual maturity on the reproductive performance of boars after sexual maturity. The results showed that the time to ejaculation was shortened by nearly 50 s, and the duration was prolonged by 30 s (*P* > 0.05) (Figure 1). Furthermore, the number of effective sperm in Fibre group boars was 16% higher than in the Control group (*P* < 0.05).

### Serum and Testis Metabolites

As shown in Table 4, dietary fibre supplementation influenced the lipid levels in the serum and testes of boars. Compared to the Control group, the CHO (*P* < 0.05) and LDL in the Fibre group were significantly decreased (*P* < 0.01), while the CHO (*P* < 0.05), TG, and LDL contents in the testis of the Fibre group were significantly higher than those in the Control group (*P* < 0.01).

**Table 4:** Effects of dietary fibre supplementation on lipid levels in serum and testis of boars.

**Items**	**Control**	**Fibre**	***P*-value**
**Serum**			
CHO (mmol/L)	1.78 ± 0.04	1.59 ± 0.08	0.048
TG (mmol/L)	0.34 ± 0.01	0.44 ± 0.04	0.376
HDL (mmol/L)	0.53 ± 0.05	0.55 ± 0.04	0.802
LDL (mmol/L)	0.73 ± 0.04	0.68 ± 0.04	0.008
**Testis**			
CHO (mmol/gprot)	0.34 ± 0.02	0.53 ± 0.01	0.041
TG (mmol/gprot)	0.71 ± 0.06	0.95 ± 0.01	0.003
HDL (mmol/gprot)	0.02 ± 0.003	0.03 ± 0.003	0.198
LDL (mmol/gprot)	0.80 ± 0.07	1.13 ± 0.06	0.002

*CHO, cholesterol; LDL, low-density lipoprotein; TG, triglyceride; HDL, high-density lipoprotein. Data were expressed as the mean ± SEM*.

### Hormone Level in Serum and Testis

As shown in Table 5, dietary fibre supplementation has great effects on hormone levels in the serum and testes of boars. Compared with the Control group, the concentrations of leptin, leptor, and LH in the serum of the Fibre group were significantly lower (*P* < 0.05). Interestingly, the hormone levels in the testicular tissue were completely different from those in serum, and the FSH and T levels in the Fibre group were higher than those in the Control group (*P* < 0.10); however, there was no significant difference in the leptin and LH levels (*P* > 0.05).

**Table 5:** Effects of dietary fibre supplementation on hormone levels in serum and testis of boars.

**Items**	**Control**	**Fibre**	***P*-value**
**Serum**			
Leptor (ng/mL)	0.61 ± 0.11	0.30 ± 0.05	0.042
Leptin (ng/mL)	0.45 ± 0.11	0.16 ± 0.03	0.024
FSH (mIU /mL)	1.60 ± 0.15	1.68 ± 0.32	0.790
LH (mIU/mL)	1.37 ± 0.12	1.01 ± 0.10	0.036
T (ng/mL)	2.99 ± 0.43	4.32 ± 0.44	0.041
**Testis**			
Leptor (ng/mgprot)	2.82 ± 0.26	3.50 ± 0.41	0.377
Leptin (ng/mgprot)	4.63 ± 0.35	6.55 ± 0.49	0.103
FSH (mIU /mgprot)	6.18 ± 0.45	8.78 ± 1.07	0.044
LH (mIU/mgprot)	3.72 ± 0.32	4.65 ± 0.60	0.333
T (ng/mgprot)	208.10 ± 11.26	275.97 ± 16.18	0.052

*FSH, follicle-stimulating hormone; LH, luteinizing hormone; T, testosterone. Data were expressed as the mean ± SEM*.

### Testicular Development and Morphology

**Figure 2:**
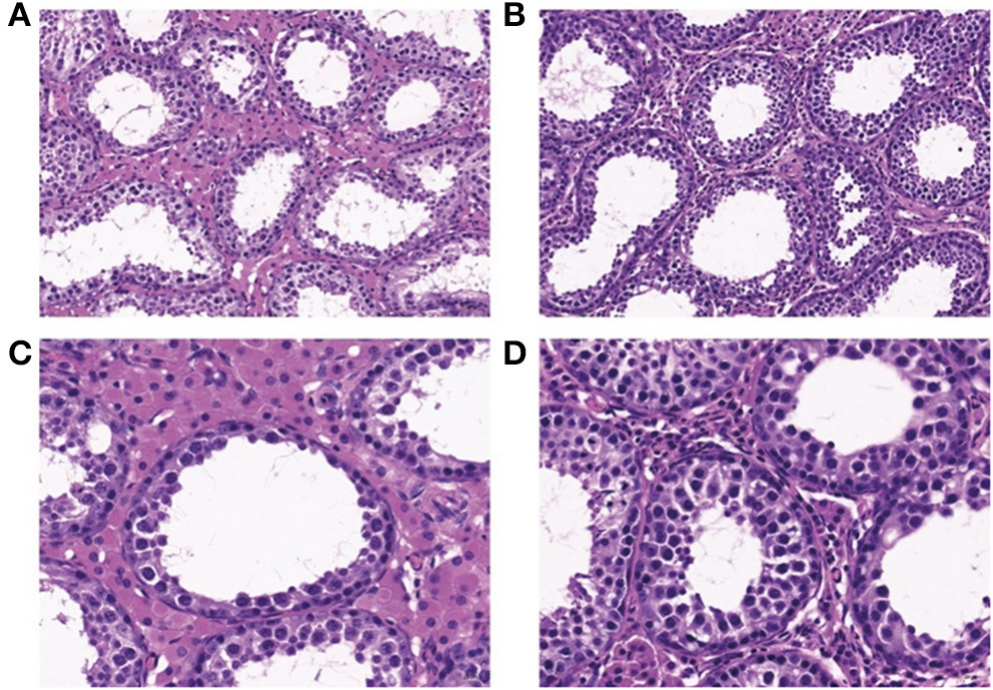
HE staining of testis tissue cross-sections of boars at 120 days. **(A, C)** Represent the Control group with a 200-fold field of vision; **(B, D)** Show the Fibre group with a 400-fold field of vision.

**Figure 3:**
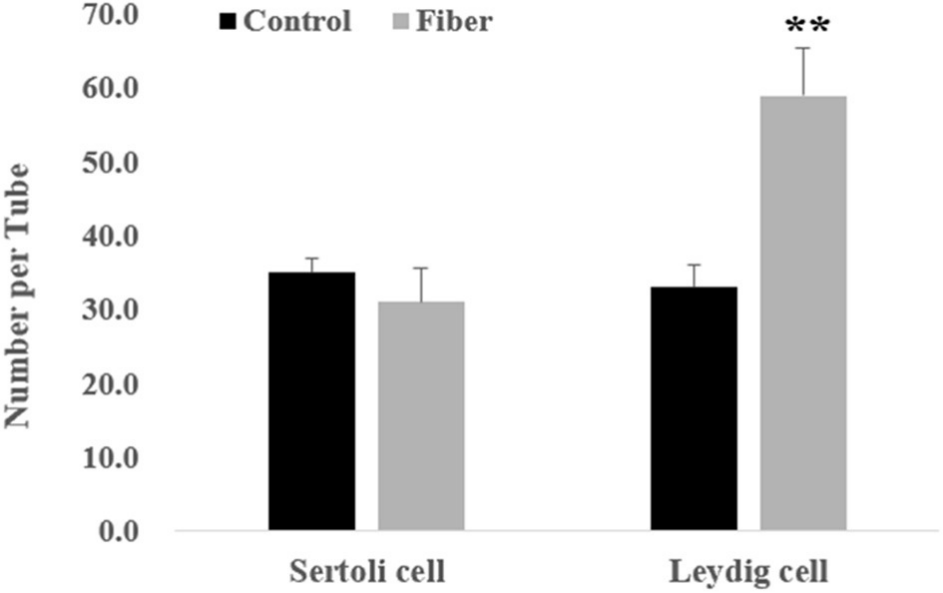
The number of germ cells in testes of boars. Leydig cell, No. of Leydig cells per tubule; Sertoli cell, No. of Sertoli cells per tubule. Data were expressed as the mean ± SEM. ^**^P < 0.01.

As shown in Figures 2A,B, at the age of 120 days, the testicular structure of boars in the Control and Fibre groups was clear and complete, but the seminiferous tubules were loosely arranged in the Control group and were tightly arranged in the Fibre group. The germ cells in the Fibre group were closely arranged, and the number of germ cell layers and germ cells in the seminiferous tubules increased (Figures 2C,D). Furthermore, no significant difference was observed in the number of Sertoli cells, whereas the number of Leydig cells in the Fibre group was significantly higher than that of the Control group (Figure 3) (*P* < 0.001).

### Relative mRNA Expression of Testis

To verify whether the fibre supplementation influenced testicular development, the mRNA expression level of proliferation and differentiation genes were analysed (Table 6). The relative expression of *AMH, AMHR2*, and *SYCP3* genes was significantly up-regulated in the Fibre group (*P* < 0.01 or *P* < 0.05).

**Table 6:** The mRNA expression of related marker genes in the testicular development of boars.

	**CT value**	**Relative expression quantity**		
**Name**	**Control**	**Fibre**	**Control**	**Fibre**
GDNF	28.49 ± 3.33	28.15 ± 2.41	1.00 ± 0.15	0.87 ± 0.12
WT1	25.73 ± 0.09	23.83 ± 0.24	1.00 ± 0.07	1.05 ± 0.09
AMH	27.98 ± 0.05	26.64 ± 0.13	1.00 ± 0.13^A^	1.87 ± 0.32^B^
AMHR2	29.05 ± 0.17	27.40 ± 0.22	1.00 ± 0.15^A^	2.32 ± 0.41^B^
AR	23.84 ± 0.49	22.59 ± 0.38	1.00 ± 0.14	1.08 ± 0.16
LEPR	26.22 ± 0.57	24.43 ± 1.03	1.00 ± 0.142	1.12 ± 0.17
LHR	23.21 ± 6.33	21.20 ± 2.64	1.00 ± 0.144	1.36 ± 0.16
HSD3B1	23.07 ± 0.29	21.72 ± 0.20	1.00 ± 0.146	1.18 ± 0.18
THP1	14.65 ± 0.45	18.37 ± 0.52	1.00 ± 0.148	0.59 ± 0.14
CDH1	22.69 ± 0.22	21.94 ± 0.35	1.00 ± 0.15	1.08 ± 0.20
SYCP3	19.14 ± 4.44	17.23 ± 3.92	1.00 ± 0.15^a^	1.36 ± 0.23^b^

*GDNF, glial cell line-derived neurotrophic factor; WT1, Wilm tumour gene1; AMH, Anti-Mullerian hormone; AMHR2, type 2 AMH receptor; AR, Androgen Receptor; LEPR, leptin receptor; LHR, luteinizing hormone receptor; HSD3B1, 3beta-hydroxysteroid dehydrogenase type 1; CDH1, E-cadherin; SYCP3, synaptonemal complex protein 3. Data were expressed as the mean ± SEM. Values with different letters differ significantly (Uppercase indicate P < 0.01, Lowercase indicates P < 0.05)*.

As shown in Table 7, compared to the Control group, the expression of steroidogenic acute regulatory (StAR) protein and the suppressors of cytokine signalling 3 (SOCS3), a leptin-inducible inhibitor, were up-regulated in the Fibre group (*P* < 0.01). Meanwhile, the expression of cytochrome P450 family 11 subfamily A member 1 (*CYP11A1*) genes was significantly up-regulated (*P* < 0.05), and the relative expression of phosphodiesterase-3B (*PDE3B*) mRNA was significantly down-regulated (*P* < 0.05).

**Table 7:** The mRNA expression of related marker genes in hormone pathway of boars.

	**CT value**	**Relative expression quantity**		
**Name**	**Control**	**Fibre**	**Control**	**Fibre**
KISS1	28.54 ± 0.05	27.51 ± 0.08	1.00 ± 0.14	0.88 ± 0.22
LEPTIN	24.51 ± 0.53	23.46 ± 0.32	1.00 ± 0.11	0.82 ± 0.19
STRA	21.26 ± 0.29	18.76 ± 0.22	1.00 ± 0.13^A^	1.85 ± 0.38 ^B^
CYP11A1	20.84 ± 0.05	19.13 ± 0.07	1.00 ± 0.15^a^	1.24 ± 0.12 ^b^
JAK2	29.49 ± 0.12	29.38 ± 0.41	1.00 ± 0.11	0.84 ± 0.13
STAT3	22.72 ± 0.33	21.48 ± 0.24	1.00 ± 0.05	1.11 ± 0.05
SOCS3	28.83 ± 0.09	27.29 ± 0.14	1.00 ± 0.08 ^A^	1.58 ± 0.18 ^B^
PDE3B	24.10 ± 0.05	22.25 ± 0.14	1.00 ± 0.05 ^a^	0.78 ± 0.16 ^b^

*KISS1, Kisspeptin gene; LEPTIN, Leptin; STRA, Steroidogenic acute regulatory protein; CYP11A1, cytochrome P450 side-chain cleavage enzyme; JAK2, Janus kinase; STAT3, Signal transducers and activators of transcription 3; SOCS3, Suppressors of Cytokine Signalling 3; PDE3B, phosphodiesterase 3B. Data were expressed as the mean ± SEM. Values with different letters differ significantly (Uppercase indicates P < 0.01, Lowercase indicates P < 0.05)*.

As shown in Table 8, the expression of the *OCCLUDINS* gene in the Fibre group was significantly up-regulated (*P* < 0.01), but the expression of CDH2 was significantly down-regulated (*P* < 0.05), and the expression of ZO-1 and CTNNB1 were not different.

**Table 8:** The mRNA expression of related marker genes in the testicular barrier of boars.

	**CT value**	**Relative expression quantity**		
**Name**	**Control**	**Fibre**	**Control**	**Fibre**
ZO1	21.09 ± 0.14	20.19 ± 0.29	1.00 ± 0.15	0.87 ± 0.22
CTNNB1	22.21 ± 0.38	21.84 ± 0.25	1.00 ± 0.14	0.87 ± 0.19
CDH2	22.69 ± 0.13	21.94 ± 0.09	1.00 ± 0.10^a^	0.83 ± 0.11^b^
OCCLUDIN	25.33 ± 0.22	23.26 ± 0.18	1.00 ± 0.20^A^	1.77 ± 0.28^B^

*ZO1, zonula occludens-1; CTNNB1, beta-catenin; CDH2, N-cadherin; OCCLUDIN. Data were expressed as the mean ± SEM. Values with different letters differ significantly (Uppercase indicates P < 0.01, Lowercase indicates P < 0.05)*.

## Discussion

In this study, growing boars were used as a model to investigate the relationship between nutrition and testicular development. Pigs are an excellent large animal model for studies of human anatomy (15). However, both humans and pigs have poor fibre digestibility. In this study, we found that a total dietary fibre (TDF) difference of approximately 1.5–2.5% had no significant effect on the growth performance of growing boars. Research has shown that fibre supplementation in weaned piglets improves their growth performance (16), is beneficial to weaned piglet health, and reduces the diarrhoea rate (17). However, Asmus et al. found that fibre supplementation did not affect the growth performance of fattening pigs, such as the average daily gain and feed-meat ratio (18), and increased the ileal protein loss rate of the castrated boars with an increase in dietary neutral detergent fibre (NDF) levels (19). Different fibre sources have different fibre compositions and fermentation capacities that affect growth and reproduction differently (20).

We further investigated the role of fibre intake on the reproductive performance of adult boars and found that it had no effect on sexual desire but increased the number of effective sperms. There is little direct evidence regarding the effect of fibre on testicular development and semen quality. Research has shown that the total motile count (TMC) is significantly higher in men who consume more fruit, nuts, and vegetables (21). Restriction of feed intake and dietary fibre supplementation can improve sperm quality in adult male rabbits (6). In fact, research on women showed that partially hydrolysed guar gum supplementation helped to improve pregnancy success in women with infertility (22) and improved the reproductive performance of sows (23). These results show that dietary fibre supplementation has a beneficial effect on the reproductive performance of both females and males.

Sperm production has been shown to be affected by testicular development and hormone secretion. The testis mainly consists of Leydig cells, Sertoli cells, and the spermatogenic cells. In males, Leydig cells are the main producers of testosterone, which is essential for sex differentiation and spermatogenesis (24). Sertoli cells also respond to the pituitary hormones, such as FSH, to begin the process of spermatogenesis. They are also known as the nursemaid cells of the primary spermatogonia (25), and androgen receptors on Sertoli cells bind testosterone to further promote sperm maturation. Our results showed that the increase in fibre intake in growing boars promoted the proliferation of Leydig cells and increased the levels of T and FSH, implying that fibre supplementation improves testicular development, which is beneficial to spermatogenesis in adulthood. However, no treatment difference was observed in free testosterone levels in healthy adult men with hyperlipidaemia after consuming a soluble fibre or insoluble fibre diet (26), while a reduction in dietary fat intake and increase in fibre intake in men resulted in the reduction of the circulating androgen levels (27). A vegan diet is associated with small but significant increases in sex-hormone-binding globulin and testosterone concentrations compared with meat-eaters in men (28).

Cholesterol, the raw material for testosterone synthesis, participates in testosterone synthesis in the Leydig cells. Extracellular LDL can be degraded into cholesterol by lysosomes (29), and HDL is transported into cells through the scavenger receptor BI (SR-BI) to directly provide raw materials for testosterone synthesis (30). Testosterone synthesis was reduced owing to the decrease in free cholesterol content in rat Leydig cells (31). In this study, we found that increased fibre intake significantly increased the boar testicular cholesterol and T levels but reduced the serum cholesterol and T levels. Studies have shown that the consumption of high-fibre diets reduces serum lipid levels and total cholesterol content in humans (32) and animals (33). Dietary fibre supplementation in pregnant sows changes the development of piglet testes and increases the levels of CHO, TG, and HDL (8). After the intragastric administration of ginger extract to rats, an increase in testosterone levels in the serum and testes is accompanied by an increase in CHO levels (34). Therefore, it is speculated that testosterone in the testis may be positively correlated with cholesterol levels, and the increase in testicular cholesterol may be beneficial to male reproduction. However, the mechanism of inconsistent cholesterol and testosterone levels in the blood and testis is not clear and is worth exploring in the future.

The serum concentrations of leptin, leptin receptor, and LH were significantly decreased in this study. The leptin receptor is expressed in human and pig seminiferous tubules, germ cells, and sperm, suggesting that leptin plays an important role in male reproduction (35). Leptin is mainly synthesised and secreted by adipocytes and plays a crucial role in energy metabolism and reproduction by binding to its receptor LepR (36). The number of sperms in male mice decreased, and the proportion of abnormal sperms increased after the intraperitoneal injection of leptin (37). Leptin levels in adult men were negatively correlated with the free and total testosterone levels. Leptin increased by 1 ng/ml, and testosterone and free testosterone decreased by 5.13 and 0.11 ng/dl, respectively (38). In summer, mammalian testosterone synthesis and semen production decreases while leptin levels increase by 180% (39, 40). The above results indicate that leptin regulates testosterone secretion and sperm production and that excessive leptin has a negative impact on spermatogenesis and sperm quality.

Testicular steroidogenic enzymes, including StAR, 3β-HSD, and CYP11a1, play important roles in testosterone synthesis (41). Hormone synthesis in the testis is mainly regulated by the Janus kinase 2-steroidogenic acute regulatory (StAR)-suppressor of cytokine signalling 3 (JAK-StAT-SOCS3) signal pathway. In this study, we found that the expression of StAR, SOCS3, and CYP11a1 was up-regulated after an increase in fibre intake. Both StAR and CYP11A1 play critical roles in testicular development and spermatogenesis by regulating androgen production and are the rate-limiting factors of steroidogenesis (42), while SOCS3 is related to androgen production and sperm production in rats (43). Research has shown that diets supplemented with fruit pulp (44) or ellagic acid (45) improve reproductive function in male rats by increasing the circulatory levels of testosterone and up-regulating steroidogenic gene expression. Furthermore, dietary fibre intake up-regulated the expression of *AMH* and *AMHR2*. The secretion of anti-Müllerian hormone (AMH) by SCs is high from early foetal life to puberty (46). In postnatal life, AMH may play a role in regulating Leydig cell proliferation and androgen biosynthesis (47). AMH signals through its dedicated type II receptor, AMHR2, which is expressed in Leydig cells (48) and germ cells (49) and plays an important role in testicular development. AMH concentrations increase during puberty and are associated with testicular width in males (50). The expression of meiotic-related protein SYCP3 and tight junction OCCLUDIN play important roles in spermatogenesis; both genes were up-regulated after fibre intake. These results confirm that dietary fibre intake improves spermatogenesis by increasing the number of Leydig cells and strengthening testicular function.

## Conclusion

In general, our results show that increasing fibre intake during the pre-puberty period increased sperm production in boars by increasing leydig cells and testosterone synthesis. These results provide important dietary guidelines for boars breeding before they reach sexual maturity.

## Data Availability Statement

The datasets presented in this study can be found in online repositories. The names of the repository/repositories and accession number(s) can be found in the article/supplementary material.

## Ethics Statement

The animal study was reviewed and approved by Animal experiments were approved by the Sichuan Agricultural University under the Ethics Approval Code SCAUAC201906-03. Written informed consent was obtained from the owners for the participation of their animals in this study.

## Author Contributions

YL, DW, and SX designed the study. JL, YL, and LL conducted the research. ZF, BF, LC, and CW analysed the data. YL and JZ wrote the manuscript. YL and DW revised the manuscript. All authors have read and agreed to the published version of the manuscript.

## Conflict of Interest

The authors declare that the research was conducted in the absence of any commercial or financial relationships that could be construed as a potential conflict of interest.

## Publisher's Note

All claims expressed in this article are solely those of the authors and do not necessarily represent those of their affiliated organizations, or those of the publisher, the editors and the reviewers. Any product that may be evaluated in this article, or claim that may be made by its manufacturer, is not guaranteed or endorsed by the publisher.
